# Retinopathy, histidine-rich protein-2 and perfusion pressure in cerebral malaria

**DOI:** 10.1093/brain/awu144

**Published:** 2014-06-11

**Authors:** Symon M. Kariuki, Charles R. J. C. Newton

**Affiliations:** 1 KEMRI-Wellcome Trust Research Programme, Kilifi, Kenya; 2 Department of Psychiatry, University of Oxford, Oxford, UK

Sir, We read with interest the detailed review by MacCormick *et al.* (2014) about the utility of retinopathy in understanding the pathogenesis of paediatric cerebral malaria. The authors conclude that the brain and retina are similar in many ways and may have similar disease processes that are relevant in cerebral malaria; for example, retinal capillary non-perfusion could be a marker for brain ischaemia and swelling often observed in cerebral malaria (Newton *et al.*, 1994). Importantly the authors point out the differences between the retina and brain, particularly arterial-venous ratios and watersheds, and metabolic peaks in childhood, all of which complicate the use of retinopathy in understanding neurovascular diseases, including cerebral malaria.

MacCormick *et al.* (2014) observe that 25% of the WHO-defined cerebral malaria may be related to other causes of coma other than *Plasmodium falciparum* malaria, as shown in an autopsy study (Taylor *et al.*, 2004). Recently, histidine-rich protein (HRP2), which reflects biomass for parasitaemia, has proved useful in the definition of cerebral malaria with both specificity and sensitivity (Hendriksen *et al.*, 2012). We think it is worth mentioning the role of HRP2 in the definition of cerebral malaria, as studies from Malawi (Seydel *et al.*, 2012), and Kenya (Kariuki *et al.*, 2014) showed that this protein is associated with many of the features of retinopathy seen in cerebral malaria; particularly, retinal whitening and haemorrhages. Furthermore, if the WHO definition of cerebral malaria includes HRP2 rather than parasitaemia, malaria-attributable fraction (MAF) improves from 88 to 93% (Kariuki *et al.*, 2014). Additionally, McCormick *et al.* (2014) state that the vessel discolouration is absent in studies that used sensitive retinal techniques, yet have been seen in 30% in some studies. Similarly, studies in Ghana and Mali did not report vessel colour changes in cerebral malaria (Schémann *et al.*, 2002; Essuman *et al.*, 2010), and in a Kenyan study (Kariuki *et al.*, 2014), HRP2 denoted a low MAF for vessel colour changes compared to other features. This raises questions as to whether this feature is a reliable marker for cerebral malaria and whether these differences are ascribed to inexperience of trained clinicians (who often do opthalmoscopic examination in resource-poor settings) or reflect regional differences in this feature. As the authors conclude, further research is warranted.

The authors omitted to discuss pressure autoregulation, and vascular response to carbon dioxide and oxygen in the brain and retina. Cerebral autoregulation seems to be impaired (Newton *et al.*, 1996) in Kenyan children with cerebral malaria, so that cerebral blood flow is likely to be determined by the cerebral perfusion pressure. It is unclear if retinal blood flow is determined by blood pressure in malaria, and raised intraocular pressure has not been reported in malaria. Many patients with cerebral malaria have hypocapnia, with deep breathing, to compensate for the metabolic acidosis. The cerebral and retinal circulations seem to have similar responses to hypocapnia (Harris *et al.*, 1998), although there may be quantitative differences. There appear to be differences in the brain and retina to arterial oxygen content, which may affect the blood flow in these organs in those patients who have severe anaemia (McLellan and Walsh, 2004).

Retinopathy is observed in a proportion of Bangladeshi adults with cerebral malaria (Abu Sayeed *et al.*, 2011), and likely represents one pathogenesis of cerebral malaria. Retinopathy-negative and retinopathy-positive children had similar odds of abnormal neurological outcomes (61.9 versus 63.0) in a Malawian study (Postels *et al.*, 2012), suggesting that both groups are clinically important. Postels *et al.* (2012) alluded to the fact that the retinopathy-negative group may have a different pathophysiological mechanism, related to the heterogeneous aetiologies observed in an autopsy study (Taylor *et al.*, 2004; Postels *et al.*, 2012), but the role of observed parasitaemia remains unclear. In fact, some authors support other pathophysiological mechanisms (e.g. inflammation) in the development of cerebral malaria (van der Heyde *et al.*, 2006), although further studies are required to understand if this has a role in retinopathy-negative cerebral malaria. Thus other surrogate biological markers such as HRP2 that can be measured easily, should be identified, since these may be more useful in terms of initiating therapeutic interventions and determine endpoints for intervention studies for both retinopathy-positive and retinopathy-negative cerebral malaria.
